# Cross-Dressing of Multiple Myeloma Cells Mediated by Extracellular Vesicles Conveying MIC and ULBP Ligands Promotes NK Cell Killing

**DOI:** 10.3390/ijms24119467

**Published:** 2023-05-30

**Authors:** Elisabetta Vulpis, Luisa Loconte, Chiara Cassone, Fabrizio Antonangeli, Giulio Caracciolo, Laura Masuelli, Francesca Fazio, Maria Teresa Petrucci, Cinzia Fionda, Alessandra Soriani, Cristina Cerboni, Marco Cippitelli, Angela Santoni, Alessandra Zingoni

**Affiliations:** 1 Laboratory Affiliated to Istituto Pasteur Italia-Fondazione Cenci Bolognetti, Department of Molecular Medicine, “Sapienza” University of Rome, 00185 Rome, Italy; elisabetta.vulpis@uniroma1.it (E.V.); luisa.loconte@uniroma1.it (L.L.); chiara.cassone@uniroma1.it (C.C.); cinzia.fionda@uniroma1.it (C.F.); marco.cippitelli@uniroma1.it (M.C.); angela.santoni@uniroma1.it (A.S.); 2 Institute of Molecular Biology and Pathology, National Research Council (CNR), 00185 Rome, Italy; fabrizio.antonangeli@cnr.it; 3 Department of Molecular Medicine, “Sapienza” University of Rome, 00161 Rome, Italy; giulio.caracciolo@uniroma1.it; 4 Department of Experimental Medicine, “Sapienza” University of Rome, 00161 Rome, Italy; 5 Department of Cellular Biotechnologies and Hematology, “Sapienza” University of Rome, 00161 Rome, Italy; 6 IRCCS Neuromed, 86077 Pozzilli, Italy

**Keywords:** extracellular vesicles, NKG2D, natural killer cells, multiple myeloma

## Abstract

Natural Killer (NK) cells are innate cytotoxic lymphoid cells that play a crucial role in cancer immunosurveillance. NKG2D is an activating receptor that binds to MIC and ULBP molecules typically induced on damaged, transformed, or infected cells. The secretion of NKG2D ligands (NKG2DLs) through protease-mediated cleavage or in an extracellular vesicle (EV) is a mode to control their cell surface expression and a mechanism used by cancer cells to evade NKG2D-mediated immunosurveillance. EVs are emerging as important players in mediating cell-to-cell communication due to their ability to transfer biological material to acceptor cells. Herein, we investigated the spreading of NKG2DLs of both MIC and ULBP molecules through the EV-mediated cross-dressing on multiple myeloma (MM) cells. We focused our attention on two MICA allelic variants, namely MICA*008 and MICA*019, representing the prototype of short and long MICA alleles, respectively, and on ULBP-1, ULBP-2, and ULBP-3. Our findings demonstrate that both ULBP and MICA ligands can be acquired from tumor cells through EVs enhancing NK cell recognition and killing. Moreover, besides MICA, EVs expressing ULBP-1 but not ULBP-2 and 3 were detected in bone marrow aspirates derived from a cohort of MM patients. Our findings shed light on the role of EV-associated MICA allelic variants and ULBP molecules in the modulation of NKG2D-mediated NK cell immunosurveillance in the tumor microenvironment. Moreover, the EV-mediated transfer of NKG2DLs could suggest novel therapeutic approaches based on the usage of engineered nanoparticles aimed at increasing cancer cell immunogenicity.

## 1. Introduction

Human natural killer receptor group 2 member D (NKG2D) is a homodimeric C-type lectin-like transmembrane receptor that is expressed on different cell populations of both the innate and adaptive immune system, such as CD8^+^ αβ T cells, γδ T cells, Natural Killer (NK) cells, and NKT cells [1,2]. The activation of the NKG2D signaling pathway is triggered by the engagement of its ligands, resulting in immune effector functions, such as cytokine production and cellular cytotoxicity [3]. NKG2D ligands (NKG2DLs) are “stress-inducible” molecules belonging to two different families in humans: the MHC class I chain-related protein A/B (MICA/B) and the UL16-binding proteins (ULBP1-6) [4]. These ligands are characterized by a high grade of polymorphism, in particular MICA, and this variability has been shown to influence the structure of the ligand and/or its affinity to NKG2D. For example, a microsatellite polymorphism in the transmembrane region of MICA results in an early stop codon and, consequently, in a truncated protein as described for the allelic variant MICA*008 [5]. Moreover, NKG2DLs can be anchored to the plasma membrane as transmembrane (TM) proteins, such as MICA, MICB, and ULBP4 or through a glycophosphatidylinositol (GPI) motif, such as ULBP-1, ULBP-3, ULBP-6, and MICA*008, while ULBP-2 and -5 could be potentially expressed as either TM or GPI [6,7].

Over the last few years, a number of studies provided evidence that the NKG2D/NKG2DL axis plays a pivotal role in the early recognition and elimination of transformed cells, even if different mechanisms of tumor immunoescape often interfere with this pathway during cancer progression [8,9]. In fact, to avoid the NKG2D-mediated recognition, cancer cells release soluble NKG2DLs through the proteolytic cleavage or exosome secretion [10], thus reducing their surface expression [11,12,13,14]. The different ways NKG2DL are anchored to the plasma membrane are the determinants to dictate which of the two mechanisms of ligand release might be more prevalent. As such, the transmembrane form of NKG2DL is mainly shed after proteolytic cleavage through metalloproteinase activity while the GPI-anchored ligands are preferentially released via exosomes [7,15,16]. Nevertheless, the presence of both GPI-anchored and transmembrane NKG2DLs on extracellular vesicles (EVs) has been described in different cellular models [16,17,18,19,20,21,22].

Extracellular vesicles are a group of bilayer membrane vesicles characterized by a heterogeneous range of sizes, distinct mechanisms of formation in cellular compartments, and release [23]. The smallest type (30–150 nm) is represented by the exosomes or small size EV (sEV) formed in the late endosomal compartment while the microvesicles or medium-sized EV (mEV) are secreted through the budding of the plasma membrane and are characterized by a size ranging from 100–1000 nm [24]; moreover, apoptotic bodies represent a third category of large EVs (>1000 nm). Once released, EVs can interact with cells, such as immune system populations present in the microenvironment or in distant sites, modulating their effector functions through a receptor-ligand engagement or delivering some biologically active molecules through cellular uptake [18,25,26].

Trogocytosis is an active process involving the exchange of proteins between cells that contact each other and, in particular, after the formation of the immunological synapse [27]. Thus, peptide-MHC complexes are transferred from antigen-presenting cells to T lymphocytes [20,21]. In addition, a number of studies have shown the acquisition of different proteins from tumors to NK cells, such as PD1 [28] as well as NKG2DLs [29,30].

A similar process is called cross-dressing and consists of a protein transfer mediated by EVs. As such, the MHC cross-dressing of APCs via EVs has been shown to be a mechanism of Ag-spreading [31]; in addition, the transfer of chemokine receptor CCR5 [32] as well as the oncogenic receptor EGFRvIII [33] and CD47 [34] allows for the propagation of a characteristic phenotype to target cells. We have recently demonstrated that the allelic variant MICA*008 can be transferred from multiple myeloma (MM)-derived EVs to the NK cell surface, promoting NK cell fratricide [18]. Nowadays, cross-dressing through EVs still remains a mechanism poorly investigated.

MM is an hemalotogic malignancy characterized by the expansion of plasma cells (PCs) in the bone marrow [35]. Among different immune effectors, a number of studies have reported that NK cells are crucial in the immunesurveillance of MM thanks to their ability to produce a plethora of chemokines and cytokines and to directly kill MM cells. To this regard, NKG2D is involved in the NK cell-mediated recognition and killing of MM cells, and for this reason, the NKG2D-NKG2DL pathway has been the subject of intense research in MM targeted therapy [35].

Herein, we investigated the spreading of NKG2DLs belonging to both MIC and ULBP families through the mEV-mediated cross-dressing on MM cells. We focused our attention on two MICA allelic variants, namely MICA*008 and MICA*019, representing the prototype of short and long MICA alleles, respectively, and on ULBP-1, ULBP-2, and ULBP-3. Our findings demonstrate that both ULBPs and MICA ligands can be acquired by tumor cells through EVs enhancing NK cell recognition and killing. Moreover, besides MICA, EVs expressing ULBP-1 were enriched in the bone marrow of MM patients.

## 2. Results

### 2.1. Surface Expression of MICA on mEVs Derived from Both MICA*008 and MICA*019 Expressing Cells

**Figure 1:**
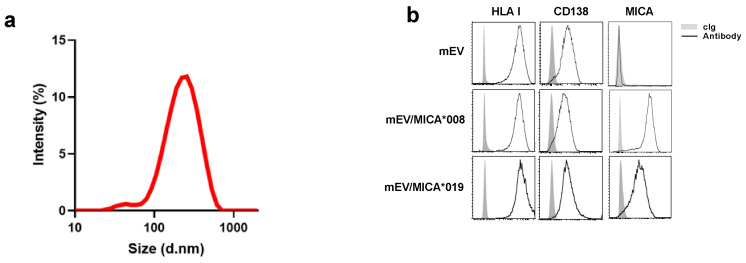
Characterization of MICA^+^mEVs. Medium-sized extracellular vesicles (mEVs) were purified from the conditioned medium derived from a Multiple Myeloma (MM) cell line ARK transfected with two MICA allelic variants namely MICA*008 or MICA*019 or with an empty vector as control. (**a**) Characterization of mEVs through DLS. (**b**) About 5–10 μg of mEVs were labeled with fluorochrome-conjugates specific monoclonal antibodies αHLA I, αCD138, and αMICA in combination with phalloidin/FITC for 60 min at room temperature. The mEVs were washed and analyzed through immunofluorescence and FACS analysis by gating on the phalloidin negative population. A representative experiment is shown.

We have recently demonstrated that the MICA allelic variant MICA*008 is exposed on the surface of both small and medium-sized EVs, released from MM cells expressing MICA*008 [18]. It is known that this allelic MICA variant is linked on the plasma membrane through a GPI anchor, and this characteristic favors its release in association with EVs [7,15]. Thus, we examined whether MICA*019, the prototype of the long allelic form of MICA with an intact transmembrane region, could be present on EV surface. We focused on medium-sized extracellular vesicles (mEVs) recovered after low speed centrifugation (13,000× *g*) isolated from ARK MM cell line (i.e., not expressing MIC ligands) transfected with MICA*008 allele, MICA*019 allele or with an empty vector [14]. Dynamic light scattering (DLS) methodology was used to evaluate EV size distribution showing that these vesicles had an average diameter corresponded to 218 ± 18 nm (Figure 1a). As previously reported, this class of medium-sized EVs was characterized by a mild expression of CD81 and CD63 tetraspanins and high expression levels of HLA I molecules [18].

As a next step, we evaluated the expression of MICA allelic variants on the surface of isolated vesicles through immunofluorescence and an FACS analysis (Supplementary Figure S1). We observed that mEVs released from both MICA*008 and MICA*019 transfected ARK MM cells were positive for both MICA allelic variants, although at different expression levels, and expressed comparable levels of HLA I as well as CD138, a marker of malignant plasma cells (Figure 1b).

### 2.2. Both MICA*008 and MICA*019 Allelic Variants Can Be Transferred from mEV to MM Cells

**Figure 2:**
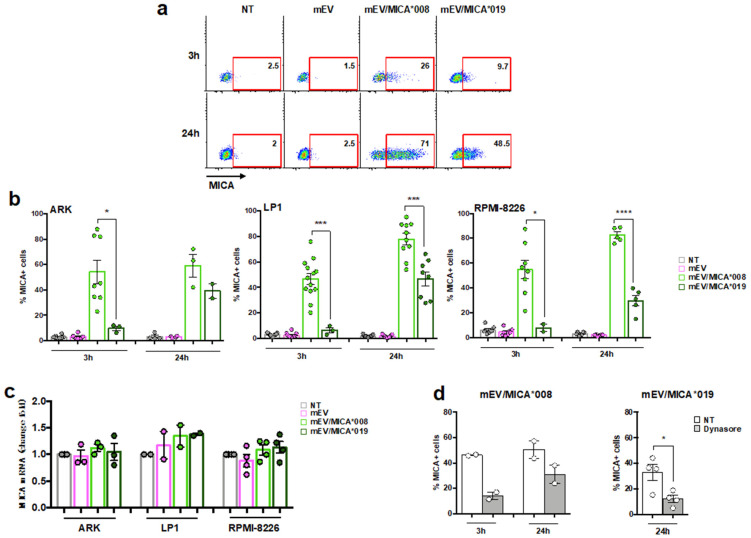
MICA can be transferred from mEV to MM cells through an endocytosis-dependent mechanism. (**a**,**b**) The human MM cell lines LP1, ARK, and RPMI-8226 were seeded at the concentration of 0.5 × 10^6^/mL in complete medium and treated for 3 and 24 h with 20 μg/mL of mEVs, mEV/MICA*008^+^, and mEV/MICA*019^+^. Cells were then harvested and stained with a specific αMICA antibody and analyzed through immunofluorescence and FACS analysis. (**a**) A representative experiment using LP1 cell line as acceptor is shown. Numbers in the boxes represent the percentage of MICA^+^LP1 cells. (**b**) The mean of the different independent experiments performed on distinct MM cell lines is shown. Values represent the percentage of MICA^+^MM cells. Statistical analysis was performed with the paired Student’s *t*-test, * *p* < 0.05, *** *p* < 0.001 and **** *p* < 0.0001. (**c**) The human MM cell lines LP1, ARK, and RPMI-8226 were treated for 24 h with mEVs as described in panel (**a**,**b**). A Real-Time PCR analysis of MICA mRNA is shown. Data, expressed as fold change, were normalized with β-actin and referred to untreated cells considered as calibrator. Values reported represent the mean of at least two independent experiments. (**d**) The LP1 cell line was pre-treated for 1 h with 10 μM of dynasore and then mEVs, mEV/MICA*008^+^, mEV/MICA*0019^+^ were added at 20 μg/mL and left for additional 3 and 24 h as indicated. The percentage of MICA^+^LP1 cells was evaluated with immunofluorescence and FACS analysis. Values represent the mean of at least two independent experiments.

A great deal of evidence described that NK cells acquire cell surface proteins, including NKG2DLs, from target cells in a direct cell contact-dependent manner [32,33]. Moreover, we have recently demonstrated that this protein transfer could also occur by EV-cell interaction. In particular, we observed that MICA*008 was transferred from EVs to the surface of NK cells through a mechanism partially dependent on NKG2D [18]. At this point, we explored whether MICA could be acquired from cancer cells in an NKG2D independent manner. To achieve this, distinct MM cell lines were treated for different times with MICA*008^+^ or MICA*019^+^ mEVs, and the percentage of MM cells expressing MICA was evaluated through immunofluorescence and an FACS analysis. We found that the MIC transfer from mEV to MM cell surface was time dependent and significantly differed between the two alleles, as the MICA*008 transfer was already evident after three hours on about 40% of MM cells whereas the MICA*019 transfer was detected after 24 h and with a reduced efficiency (Figure 2a,b). To confirm that MICA expressed on the surface of MM cell lines derived from the effective transfer of MICA as protein from mEVs to the MM cell surface and was not due to a neo-induction on receiving cells or possible transfer of mRNA contained in vesicle cargo, we analyzed MICA mRNA in MM cells upon treatment with both MICA*008 and MICA*019 mEVs. As shown in Figure 2c, MICA mRNA levels were not affected after incubation with mEVs expressing MICA or not, indicating that the increased expression of the MICA protein was attributable to the transfer from mEVs to acceptor cells.

The capability NKG2DL transfer was also assessed on mEVs derived from SKO-007(J3) MM cell line that constitutively expresses MICA (i.e., MICA*008 allelic variant). As shown in Supplementary Figure S2, mEVs derived from SKO-007(J3) expressed MICA on their surface and were able to transfer this ligand on target cells, suggesting that NKG2DL cross-dressing mediated by EVs represents a phenomenon that might occur in physiological and pathological conditions.

As a next step, considering that MM cells do not express NKG2D, we investigated the mechanism underlying the transfer of MICA from mEV to the MM cell surface in the absence of its cognate receptor. For this reason, MM cells were pre-treated with dynasore, an inhibitor of dynamin activity, which prevents membrane fission during clathrin-mediated endocytosis, and then, mEVs were added. Interestingly, our findings showed a strong reduction in the percentage of MICA^+^MM cells upon incubation with both MICA*008- and MICA*019-expressing mEVs (Figure 2d).

Furthermore, we investigated whether clathrin-mediated endocytosis was also involved in the uptake of mEVs by MM cells. Preliminary kinetic experiments demonstrated an increase in the uptake process with the augmentation of the incubation time until reaching a plateau after an overnight incubation. Thus, MM cells were pre-treated with dynasore before the incubation with PKH26^+^ mEV expressing MICA or not, and the percentage of PKH26^+^ MM cells was evaluated. Our data demonstrate that dynasore significantly blocks mEV uptake by MM cells both after 3 and 24 h (Supplementary Figure S3).

All together, these data indicate that clathrin-mediated endocytosis is involved either in the mEV uptake or in the transfer of both MICA allelic variants from mEVs to the MM cell surface.

### 2.3. MICA Transferred on MM Cells Increases Their Susceptibility to NK Cell Lysis

**Figure 3:**
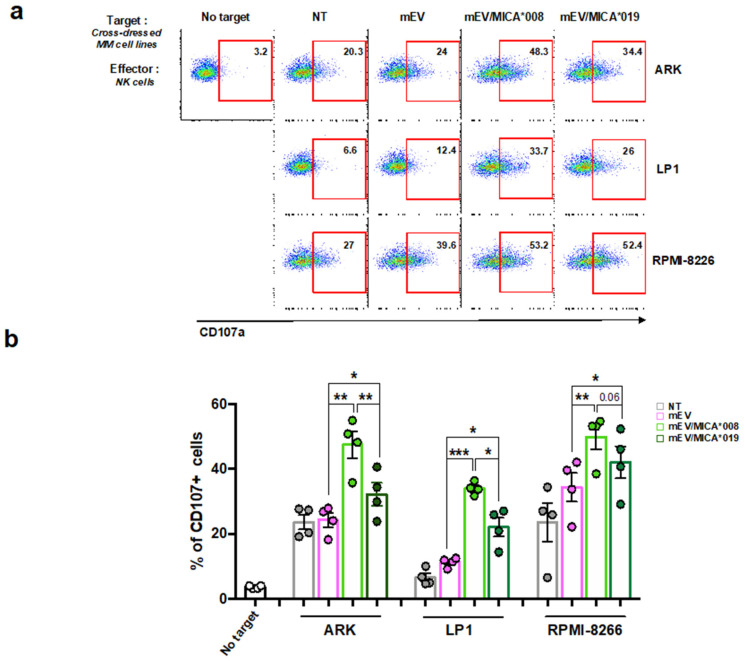
MM cells dressed with MICA become susceptible to NK cell lysis. The MM cell lines LP1, ARK, and RPMI-8226 were treated for 24 h with 20 μg/mL of mEVs, mEV/MICA*008^+^, mEV/MICA*0019^+^. Cells were harvested and used as target cells in a NK cell-degranulation assay. Briefly, EV-treated MM cells (target, T) were co-cultured with polyclonal human CFSE^+^NK cells (effector, E) for 2 h using an E:T ratio 2:1. Cells were harvested and the expression of the degranulation marker CD107a was evaluated on CFSE^+^NK cells through immunofluorescence and FACS analysis. (**a**) A representative experiment is shown. Numbers in the boxes represent the percentage of CD107a^+^ NK cells. (**b**) Data are expressed as the percentage of CD107a^+^ NK cells and represent the mean of at least four independent experiments. Statistical analysis was performed with the paired Student’s *t*-test, * *p* < 0.05, ** *p* < 0.01 and *** *p* < 0.001.

We next investigated whether the acquisition of MICA on MM cells from mEVs was able to enhance their susceptibility to NK cell cytotoxicity. At first ARK, LP-1 and RPMI-8226 cell lines were incubated with MICA^+^EVs to allow the transfer of MICA to the surface of MM cells, and then, these cells were used as targets in a degranulation assay. As shown in Figure 3a,b, NK cell degranulation significantly increased in the presence of MM cells dressed with either MICA*008 and MICA*019 compared to cells untreated or treated with empty vesicles. Notably, MICA*008-treated MM cells were the most susceptible to NK cell lysis (Figure 3a,b).

These results demonstrate that MICA allelic variants maintain their biological function upon the transfer to MM cell surface allowing a better recognition by NK cells.

### 2.4. MM Cells Acquire ULBP Molecules from mEVs and Become Sensitive to NK Cell Attack

**Figure 4:**
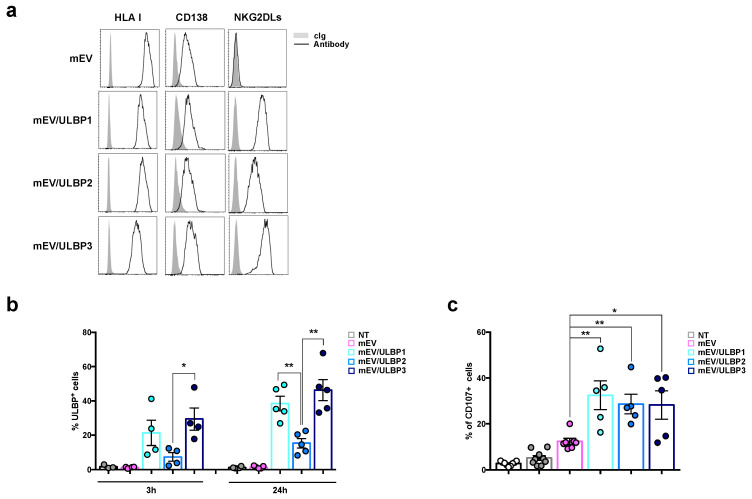
Ligands of ULBP family are expressed on mEV surface and can be transferred to MM cells, increasing their susceptibility to NK cell lysis. (**a**) About 5–10 μg of mEVs were labeled with fluorochrome-conjugates specific monoclonal antibodies αHLA I, αCD138, αULBP-1, αULBP-2, and αULBP-3 in combination with phalloidin/FITC for 60 min at room temperature. The mEVs were washed and analyzed through immunofluorescence and FACS analysis by gating on the phalloidin negative population. A representative experiment is shown. (**b**) The human MM cell line LP1 was seeded at the concentration of 0.5 × 10^6^/mL in complete medium and treated for 3 and 24 h with 20 μg/mL of mEVs, mEV/ULBP1^+^, mEV/ULBP2^+^, and mEV/ULBP3^+^. Cells were then harvested and stained with a specific αULBP-1, αULBP-2, and αULBP-3 antibody and analyzed through immunofluorescence and FACS analysis. Values represent the percentage of ULBP^+^ LP1 cells. The mean of different independent experiments is shown. (**c**) The MM cell line LP1 was treated for 24 h with 20 μg/mL of mEVs, mEV/ULBP1^+^, mEV/ULBP2^+^, and mEV/ULBP3^+^. Cells were harvested and used as targets in an NK cell degranulation assay as described in the legend of Figure 3. Data are expressed as the percentage of CD107a^+^ NK cells and represent the mean of at least five independent experiments. Statistical analysis was performed with the paired Student’s *t*-test, * *p* < 0.05 and ** *p* < 0.01.

After analyzing the effect of MICA spreading through EVs on acceptor cells, we investigated whether NKG2DLs belonging to ULBP family could also share the same characteristics. To this end, the MM cell line ARK, which does not express endogenous ULBP-1, ULBP-2, and ULBP-3, was stably transduced with cDNA encoding these ligands and ULBPs^+^mEVs were further isolated from the conditioned culture media. The expression of ULBP molecules on the mEV surface was assessed with immunofluorescence and an FACS analysis as shown in Figure 4a. Interestingly, ULBP1 and ULBP3, which are characterized by GPI anchorage on the plasma membrane, were expressed at higher levels on the surface of mEVs compared to ULBP2, a ligand with the “classical” transmembrane region. Like MICA*008 and MICA*019 expressing mEVs, these vesicles expressed similar amounts of HLA I and CD138, too (Figure 4a). As a next step, we assessed whether these NKG2DLs could move from mEVs to the MM cell surface. To this purpose, MM cells were incubated with ULBP^+^mEVs for 3 h and 24 h. Similar to MICA alleles, GPI-anchored ULBPs (i.e., ULBP1 and ULBP3) were transferred from vesicles to the MM cell surface more efficiently compared to ULBP2 in a time dependent manner (Figure 4b). Significative differences were observed between ULBP-2 cross-dressed MM cells with respect to ULBP-3 and ULBP-1 after 3 and 24 h (Figure 4b). Moreover, MM cells dressed with ULBPs were used as target cells in a degranulation assay to evaluate their ability of activating NK cells. As shown in Figure 4c, the transfer of these NKG2DLs on MM cells significantly increased their ability to trigger NK cell degranulation at comparable levels (Figure 4c).

**Figure 5:**
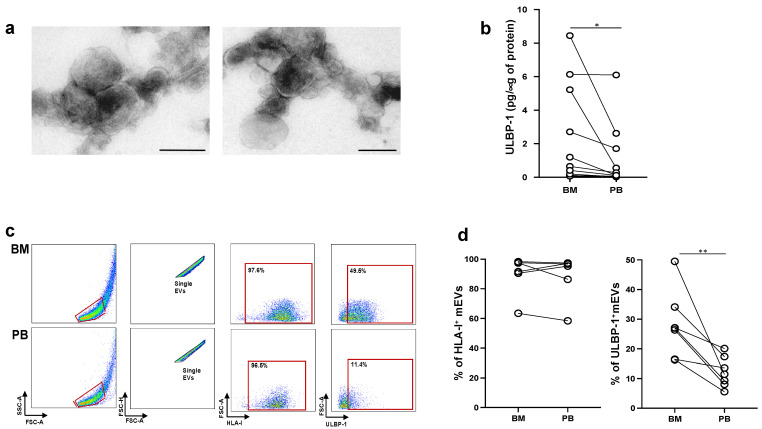
ULBP1^+^ mEVs are enriched in the bone marrow of MM patients. mEVs were isolated from peripheral blood (PB) or bone marrow (BM) aspirates of a cohort of MM patients. (**a**) Ultrastructural analysis of a representative sample. Bar corresponds to 200 nm. (**b**) Lysates derived from mEVs isolated from both BM and PB of MM patients were assessed for the presence of ULBP-1 using a specific ELISA. Values are represented as the amount of ULBP-1/μg of proteins and are derived from a cohort of nine MM patients. (**c**,**d**) Immunofluorescence and FACS analysis of mEVs using antibodies against HLA-I and ULBP-1; (**c**) dot plots showing the gating strategy related to a representative patient. The phalloidin negative population was gated and the doublets were removed by plotting FSC-H and FSC-A.The percentage of ULBP-1^+^mEVs was evaluated by gating gated on HLA-I^+^ mEVs as indicated. (**d**) Data derived from a cohort of six patients. Statistical analysis was performed with the paired Student’s *t*-test, * *p* < 0.05, and ** *p* < 0.01.

The presence of soluble NKG2DLs in the sera of cancer patients is correlated with the progression in several tumor models, including MM. However, little is known about ligands associated to EVs and their potential role in the modulation of NKG2D-dependent functions. As such, since MICA^+^EVs have been detected in the bone marrow microenvironment of MM patients [18], we further explored whether ULBP-containing mEVs were also present. To this aim, mEVs were purified from plasma derived from peripheral blood or bone marrow aspirates of MM patients (Supplementary Figure S4). As shown in Figure 5a, an ultrastructural analysis through TEM confirmed that purified EVs contained nano-sized vesicles. In addition, the size distribution of the mEV population showed an average diameter that corresponded to 140 ± 20 nm (Supplementary Figure S5). At first, mEV preparations were lysed and assessed for the presence of ULBP1-3 by ELISA. Our findings showed that ULBP1^+^mEVs were mainly found in the BM-derived plasma when compared to PB of the same patients (Figure 5b); in contrast, neither ULBP-2 or ULBP-3 expressing mEVs were detected. Moreover, by immunofluorescence and FACS analysis, we investigated the surface expression of ULBP-1 on these mEVs. As shown in Figure 5c,d, although the levels of HLA I were comparable in the two pools of mEVs, ULBP-1 was mainly located on mEV-derived from BM, thus confirming the results obtained through ELISA.

## 3. Discussion

Numerous studies over the last few years unraveled a pivotal role of tumor-derived EVs in the modulation of both innate and adaptive immune responses [19,36,37]. Our attention has focused on NK cells that are able to directly recognize and eliminate tumor cells thanks to a plethora of distinct activating receptors. Among them, NKG2D is considered the master activating NK cell receptor able to bind ligands that are only expressed at low levels in normal cells but can be upregulated by a cellular stress response [9,38].

Herein, we investigated the immunomodulatory properties of NKG2D ligands belonging to the MIC and ULBP families associated with EVs and provide novel evidence that these ligands can be transferred to target cells keeping their biological activity.

A typical strategy adopted by tumors to escape NKG2D-mediated immunosurveillance relies on the release of NKG2D ligands as soluble molecules through proteolytic cleavage or in association with vesicles. Many experimental results have demonstrated the presence of soluble NKG2D ligands in the serum of patients with various types of cancer [39,40,41], including MM, in which soluble MICA has been correlated with a progression of the disease [42,43,44]. Interestingly, our previous study has described the presence of MICA-expressing vesicles in plasma obtained from bone marrow aspirates of MM patients [18], and here, our findings also reported the detection of ULBP1^+^EVs suggesting that tumor microenvironment is enriched with potentially immunomodulatory vesicles.

The interaction between NKG2D and its ligands exposed on EVs determines a conspicuous reduction of NKG2D on the cell surface that is often associated with an impairment of NKG2D-mediated functions as reported in various experimental models [15,16,18,20,21,22]. However, it should be considered that the ability of vesicle-associated NKG2D ligands to be transferred to other cells is still a poorly studied phenomenon. Our results provide novel evidence that both MICA allelic variants (i.e.,: MICA*008 and MICA*19) and ULBP-1, -2, and -3 molecules can be transferred from vesicles to MM acceptor cells. Our findings demonstrate that this transfer occurs by a dynamin-dependent endocytosis-mediated mechanism that is also involved in vesicle uptake by acceptor cells. It is known that mEVs can be internalized via several processes, such as direct fusion with the target cell, endocytosis, phagocytosis, and micropinocytosis [45]. Collectively, the results we obtained establish that clathrin-dependent endocytosis is a process used by MM cells to capture microvesicles and acquire molecules from them.

Since MICA is a highly polymorphic molecule [4,46], we studied two allelic variants of MICA (i.e., MICA*008 and MICA*019), which represent the prototype of the short or long form of MICA, respectively. In this regard, MICA*008 has a shorter transmembrane region and is associated with the plasma membrane with a GPI anchor. Ashiru and colleagues demonstrated how the differences at the level of the TM region are responsible for the different location at the level of membrane microdomains and also for a different release by tumor cells [15]. Similarly, ULBP-1 and -3 molecules are membrane anchored through the GPI in contrast to ULBP-2. Our results confirm that GPI-associated ligands (MICA*008, ULBP-1, ULBP-3) are expressed at higher levels on vesicles than the other transmembrane-type ligands (MICA*019, ULBP-2), and interestingly, the transfer of GPI-associated ligands occurs more efficiently than the other transmembrane types.

So far, the transfer of NKG2D ligands has been demonstrated through the mechanism of trogocytosis, both in a mouse model [47] and in human cells [30]. Trogocytosis is a typical mechanism of hematopoietic cells by which an exchange of molecules associated with the plasma membrane takes place between cells that come into contact. Furthermore, recent data produced in our laboratory demonstrated that the MICA*008 allelic variant can be transferred from vesicles to NK cells promoting fratricide [18]. The transfer of molecules from vesicles to acceptor cells has been shown in the context of the immune response in the case of exosome-associated MHC/peptide complexes produced either by mature DC cells or by tumor cells. In this context, Raposo and colleagues had shown that these exosomes were capable of activating T lymphocytes in an antigen-dependent manner [48]. Moreover, a number of studies reported that EV-induced antigen cross-dressing was implicated in the regulation of alloantigen recognition and allograft rejection [31,49,50]. Interestingly, other mechanisms of an EV-mediated exchange of surface molecules between cells are emerging. To this regard, recent findings demonstrated that CD47 cross-dressing led to an impairment of phagocytosis without transmitting cell death signals by extracellular vesicles [34].

Finally, we investigated the functional significance of NKG2D ligand transfer on MM cells. In particular, we assessed whether the acceptor cell after ligand acquisition by vesicles could be recognized and lysed by NK cells through an NKG2D-mediated cytotoxic mechanism. Our results demonstrate that MM cells that acquire NKG2D ligands by vesicles display an increased ability to trigger NK cell cytotoxicity. Collectively, our observations suggest that the diffusion of NKG2D ligands in the tumor microenvironment and uptake of these ligands by tumor cells could promote NK cell activation by optimizing the immune response against the tumor.

In this scenario, EVs represent a vehicle for transporting and diffusing ligands in the tumor microenvironment. The spreading of NKG2D ligands through EVs could be considered a double-edged sword for MM cell immunosurveillance because on the one hand it could facilitate immunoevasion by inducing NKG2D downmodulation and NK cell fratricide but on the other hand it could abet NK cell recognition and killing of tumor cells cross-dressed with NKG2DLs.

## 4. Materials and Methods

### 4.1. Antibodies and Reagents

Anti-ULBP1/APC (clone 170818), anti-ULBP2/APC (clone 165903), and anti-ULBP3/APC (clone 166510) were from R&D Systems (Minneapolis, MN, USA). Anti-HLA ABC/BV421 (clone G46-2.6), anti-MICA/BV421 (clone 159227), anti-CD138/FITC (clone MI15), anti-CD107a/APC (clone H4A3), anti-NKG2D/APC (clone 1D11), anti-CD3/BV510 (clone SK7), anti-CD56/PE (clone NCAM16.2), isotype control IgG/APC (clone MOPC-21), isotype control IgG/BV421 (clone X40), and isotype control IgG/FITC (clone MOPC-21) were all from BD Biosciences (San Jose, CA, USA). Other reagents used were Phalloidin/FITC and Lipofectamine 2000 from Invitrogen (San Diego, CA, USA), CFSE, dynasore, PKH26, puromycin, polybrene, ampicillin, trypan blue (all from Sigma-Aldrich, St Louis, MO, USA), and recombinant human IL-2 from PeproTech (London, UK).

### 4.2. Cell Lines and Human Polyclonal NK Cell Preparations

The human MM cell lines LP-1, ARK, and RPMI-8226 were cultured in RPMI 1640 medium (EuroClone, Milan, Italy) supplemented with 10% fetal bovine serum (FBS) (GIBCO, Life Technologies, Gaithersburg, MD, USA). The cDNA encoding MICA*008 and MICA*019 were cloned in the pMSCV retroviral vector and used to transduce the human multiple myeloma cell line ARK, as previously reported. The pMX retroviral vectors containing cDNA encoding the human ULBP1, ULBP2, and ULBP3 sequences were kindly provided by Prof. Lewis L. Lanier (University of California, San Francisco, CA, USA) and were used to transduce the human MM cell line ARK. The ARK transfectants, the human chronic myeloid leukemia cell line K562, and the RPMI 8866 cell line were maintained in RPMI 1640 medium supplemented with 10% FBS. The human NK cell line NKL was cultured in RPMI 1640 medium supplemented with 10% FBS and recombinant human IL-2 (200 U/mL). All cell lines were mycoplasma free (EZ-PCR Mycoplasma test kit; Biological Industries, Cromwell, Connecticut). Human peripheral blood mononuclear cells (PBMCs) were obtained from healthy donors. Polyclonal NK cell cultures were obtained by co-culturing PBMC (1 × 10^5^ cells/mL) with irradiated (3000 rad) RPMI 8866 cells (1 × 10^5^ cells/mL) for 10 days at 37 °C in a humified 5% CO_2_ atmosphere and were routinely 90% CD16+, CD56+, CD3−, as assessed with immunofluorescence and cytofluorimetric analysis [51,52].

### 4.3. Virus Production and In Vitro Transduction

For retrovirus production, the Phoenix retrovirus packaging cell line HEK293 was transfected with pMXpie or pMX/ULBP-1, pMX/ULBP-2, pMX/ULBP-3, and the packaging vectors (pMCMVgag-pol and pMD2.G) using Lipofectamine 2000. After 48 h, virus containing supernatants were harvested, filtered, and used for infection as follows: 1 mL viral supernatant containing Polybrene (4–8 μg/mL) was used to infect 3 × 10^5^ ARK cells for 2 h. Two infection cycles were performed. After 72 h, infected cells were grown in selection media containing puromycin at 1 μg/mL.

### 4.4. Extracellular Vesicle Purification

Serum depleted from extracellular vesicles was obtained after centrifugation of FBS at 100,000× *g* for 2–3 h in a Beckman ultracentrifuge (Beckman Coulter, Brea, CA, USA. One hundred fifty ARK cells transduced with pMSCV, MICA*008, MICA*019 [14], pMXpie, ULBP-1, ULBP-2, and ULBP-3 were cultured at high density (7 × 10^6^ cells/mL) in 25 mL of RPMI 1640 supplemented with 10% of EV-free FBS and antibiotics in a bioreactor (SARSTEDT, Numbrecht, Germany) at for 48–72 h. The mEV purification was performed as previously reported with some modifications [18]. Briefly, cells were harvested via centrifugation at 300× *g* for 10 min. Cell-free supernatants were then centrifuged at 2000× *g* for 20 min to remove cells debris, followed by centrifugation at 13,000× *g* for 40 min to recover medium-sized EVs. The resulting pellet was washed in a large volume of cold PBS and centrifuged again at 13,000× *g* for 40 min at 4 °C. Finally, mEVs were resuspended in PBS for further analyses and functional studies. For uptake experiments, about 100 μg of mEVs diluted in PBS were incubated with the red fluorescent dye PKH26. The mEVs were washed twice with PBS via centrifugation at 13,000× *g* for 40 min. PKH26-labeled mEVs were diluted with PBS and used for uptake experiments. The mEVs were isolated from peripheral blood (PB) or bone marrow (BM) aspirates of MM patients as previously described [18]. Briefly, plasma samples were collected through centrifugation of bone marrow aspirates and peripheral blood samples at 1400 rpm for 10 min. Plasma samples were further diluted (1:2) in PBS and centrifuged at 2000× *g* for 20 min at 4 °C. The mEVs were then recovered through collection of the pellet obtained after centrifugation at 12,000× *g* for 40 min at 4 °C. The resulting pellet was washed in cold PBS and centrifuged twice at 12,000× *g* for 40 min at 4 °C. The mEVs were resuspended in PBS for further analyses.

### 4.5. Ultrastructural Analysis

Transmission electron microscopy (TEM) of mEVs was performed as follows: briefly, mEVs were fixed in 2% PFA and adsorbed on formvar-carbon-coated copper grids. The grids were then incubated in 1% glutaraldehyde for 5 min, washed with deionized water eight times, and then negatively stained with 2% uranyl oxalate (pH 7) for 5 min and methyl cellulose/uranyl for 10 min at 4 °C. Excess methyl cellulose/uranyl was blotted off, and the grids were air-dried and observed with a TEM (Philips Morgagni268D) at an accelerating voltage of 80 kV. Digital images were taken with Mega View imaging software (8.0).

### 4.6. Size Experiments

Dynamic light scattering (DLS) experiments were performed to measure mEV size. All the measurements were made at 25 °C on a Zetasizer Nano ZS90 spectrometer (Malvern, UK) equipped with a 5 mW HeNe laser (wavelength λ D 632.8 nm) and a non-invasive back-scattering optical setup (NIBS). For each sample, the detected intensity was processed by a digital logarithmic correlator, which computes a normalized intensity autocorrelation function. Then, the distribution of the diffusion coefficient D was obtained using the CONTIN method. An amount of 63 D was converted into an effective hydrodynamic diameter DH through the Stokes–Einstein equation: DH D KBT/(3 phD), where KBT was the system’s thermal energy and h represented the solvent viscosity. Solvent-resistant micro cuvettes (ZEN0040, Malvern, Herrenberg, Germany) were used for experiments with a sample volume of 40 μL. The mEV size distribution was calculated by a recently proposed DLS-based non-invasive tool.

The size distribution of mEVs isolated from patients’ plasma was performed using a ViewSizer^TM^ 3000 (HORIBA Instruments incorporated, Irvine, CA, USA).

### 4.7. RNA Isolation, RT-PCR, and Real-Time PCR

Total RNA from human primary purified NK cells was extracted using Total RNA Mini Kit (Geneaid, New Taipei City, Taiwan). Total RNA (100 ng–1 μg) was used for cDNA first-strand synthesis using oligo-dT (Promega, Madison, WI, USA) in a 20 μL reaction volume. Real-time PCR was performed using the ABI Prism 7900 Sequence Detection system (Applied Biosystems, Foster City, CA, USA). cDNAs were amplified in triplicate with primers for MICA (Hs00792195_m1) and human β-actin (Hs99999903_m1), all conjugated with fluorochrome FAM (Applied Biosystems). The cycling conditions were: 50 °C for 10 min, followed by 40 cycles of 95 °C for 30 s, and 60 °C for 2 min. Data were analysed using the Sequence Detector v1.7 analysis software (Applied Biosystems). The level of gene expression was measured using threshold cycle (Ct). The Ct was obtained by subtracting the Ct value of the gene of interest from the housekeeping gene (b-actin) Ct value. In the current study, we used Ct of the untreated sample as the calibrator. The fold change was calculated according to equation ΔΔCt, where ΔCt was the difference between Ct of the sample and the Ct of the calibrator (according to the formula, the value of the calibrator in each run is 1).

### 4.8. ELISA

The mEV samples were lysed in 1× RIPA lysis buffer (1% NP-40, 0.1% SDS 50 mM Tris-HCl pH 7.4, 150 mM NaCl, 0.5% sodium deoxycholate, 1 mM EDTA) plus complete protease inhibitor mixture (Sigma-Aldrich, St. Louis, MO, USA) and phosphatase inhibitors. Protein concentration was determined with the Bio-Rad Protein Assay (BPA). An amount of 25 μg of mEV extract were used for the for the detection of ULBP1-3 with ELISA kits for sULBP1, sULBP2, and sULBP3 (all from R&D Systems, Minneapolis, MN, USA).

### 4.9. Flow Cytometry of Cells and EVs

To evaluate cross-dressing, MM cell lines LP-1, ARK, and RPMI-8226, were labelled with fluorochrome conjugated mAbs anti-MICA, anti-ULBP1, anti-ULBP2, anti-ULBP3, or with the respective isotype control Ig for 25 min at 4 °C. Imunofluorescence and FACS analysis of mEVs was performed as previously described [18]. Briefly, about 5–10 μg of mEVs were labelled with anti-CD138, anti-MICA, anti-ULBP1, anti-ULBP2, an anti-ULBP3 in combination with anti-HLA I and Phalloidin/FITC for 60 min at room temperature with gentle tilting; mEVs were then washed using cold PBS twice by centrifugation at 13,000 in PBS before FACS acquisition. The size of mEVs was estimated by comparing the forward scatter signals with those of reference microspheres obtained from flow cytometry sub-micron particle size reference kit (Thermo Fisher Scientific, Waltham, MA, USA) as previously shown [18,53] and as shown in Supplementary Figure S1.

### 4.10. Extracellular Vesicles Uptake

An amount of 20 μg/mL of PKH26-labeled mEVs were incubated with LP-1 cells for 3 h or 24 h. Cells were collected, washed with PBS, and samples were analysed through immunofluorescence and FACS analysis. In a set of experiments, LP-1 cells were pre-treated for 1 h with dynasore and then incubated for 3 h or 24 h with PKH26-labeled mEVs.

### 4.11. NK Cell Stimulation and Degranulation

For the functional studies, NKL cells were starved for 16–18 h in 2% FBS medium without IL-2 and then seeded at 3 × 10^6^ cell/mL in complete medium and stimulated for different times with 20 μg/mL of mEVs before the assay. In some experiments, polyclonal NK cells were plated at 2 × 10^6^ cell/mL in complete medium and treated for 24 h with 20 μg/mL of mEVs. Degranulation assay was performed as described previously [14].

### 4.12. FACS Analysis

Samples were acquired using a FACSCanto (BD Biosciences, San Jose, CA, USA). Data analysis was performed using the FlowJo program (version 10.8.2).

### 4.13. Statistics

Error bars represent SD or, where indicated, SEM. Statistical analysis was performed with the Student’s paired test; * *p* < 0.05, ** *p* < 0.01, *** *p* < 0.001, **** *p* < 0.0001.

## Data Availability

Not applicable.

## Supplementary Materials

The supporting information can be downloaded at: https://www.mdpi.com/article/10.3390/ijms24119467/s1.
