# Supplementary material for: Cross-Dressing of Multiple Myeloma Cells Mediated by Extracellular Vesicles Conveying MIC and ULBP Ligands Promotes NK Cell Killing
Source: Int J Mol Sci. 2023 May 30;24(11):9467. doi: 10.3390/ijms24119467 (PMC10253317; doi:10.3390/ijms24119467)
Supplement: Supplementary file 1 [file ijms-24-09467-s001.zip › ijms-2367937-supplementary.pdf]

a

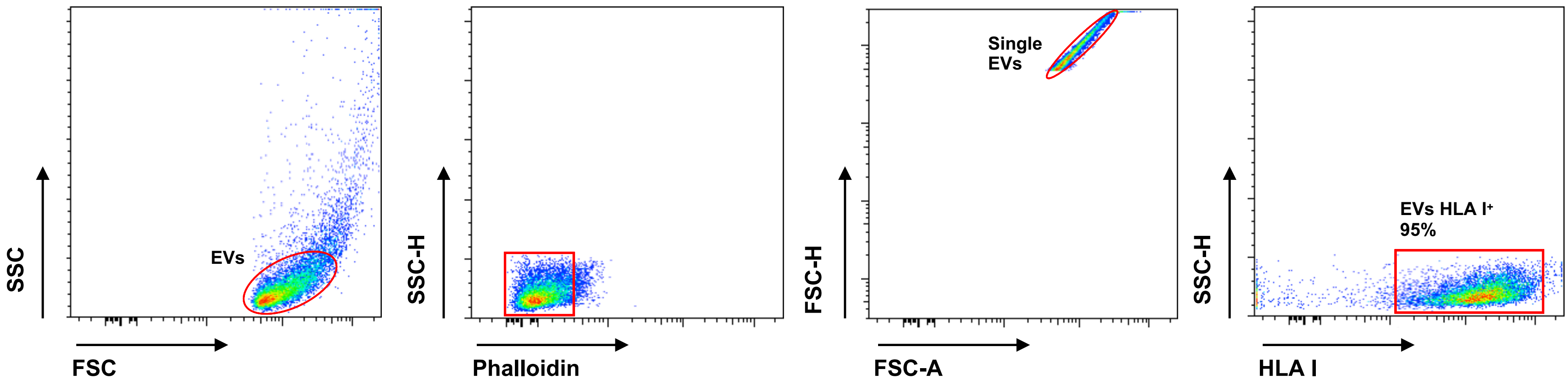

b

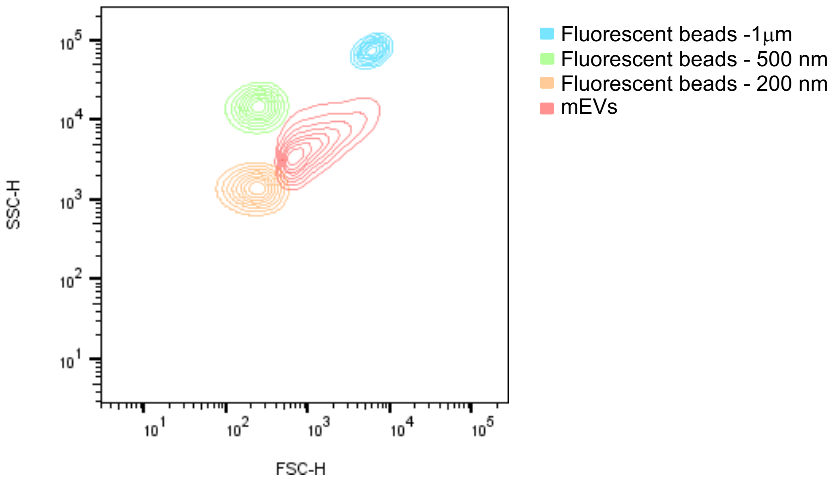

Supplementary Figure S1. Gating strategy of mEVs isolated from MM cell line ARK.

About 1-5µg of mEVs were labelled with FITC/Phalloidin in combination with specific antibodies. **a)** Dot plots showing the gating strategy. The phalloidin negative population was gated and the doublets were removed by plotting FSC-H and FSC-A; HLA I+ mEVs were gated and on this population the expression of NKG2DLs and CD138 was evaluated. **b)** Fluorescent beads of defined size (i.e: 200 nm, 500 nm and 1µM) were used to visualize the size of the selected mEV population. A representative experiment is shown.

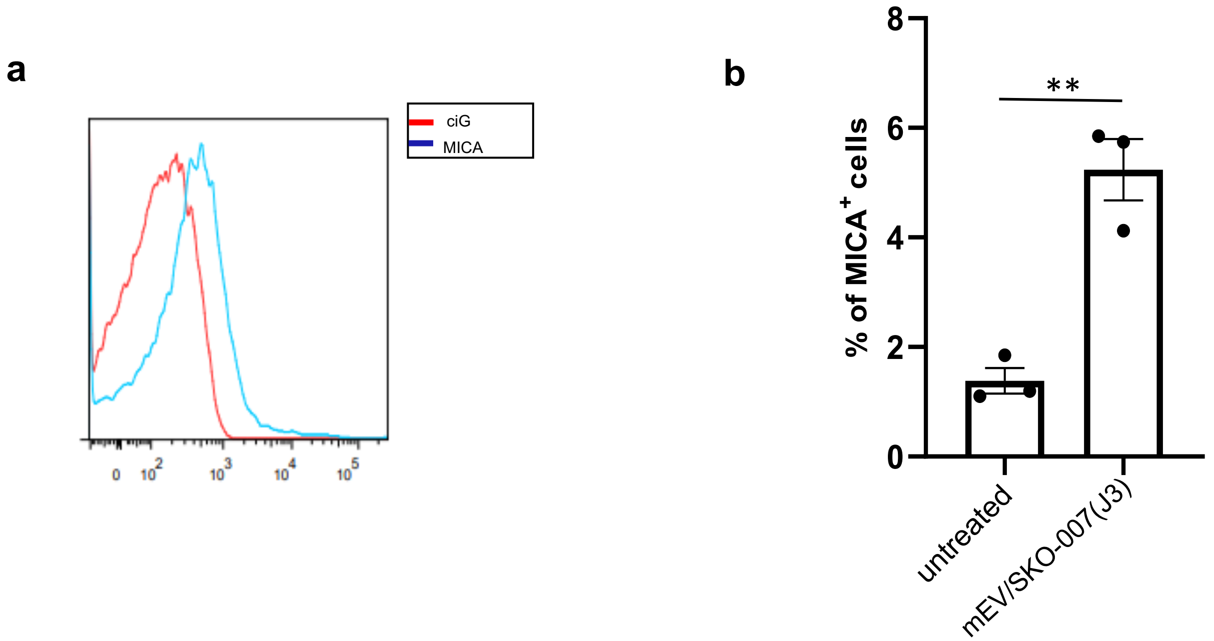

Supplementary Figure S2. mEVs derived from SKO-007(J3) MM cell line transfer MICA on target cells. a) About 5–10 µg of mEVs derived from the conditioned medium of SKO-007(J3) were labeled with fluorochrome-conjugate αMICA mAb or with an isotypic cIgG in combination with phalloidin/FITC for 60 min at room temperature. mEVs were washed and analyzed through immunofluorescence and FACS analysis by gating on the phalloidin negative population. A representative experiment is shown. b) LP1 cells were incubated with 20µg/ml of mEVs for 18 hrs; cells were harvested and stained with αMICA mAb and analyzed through FACS. The percentage of MICA positive cells is shown.

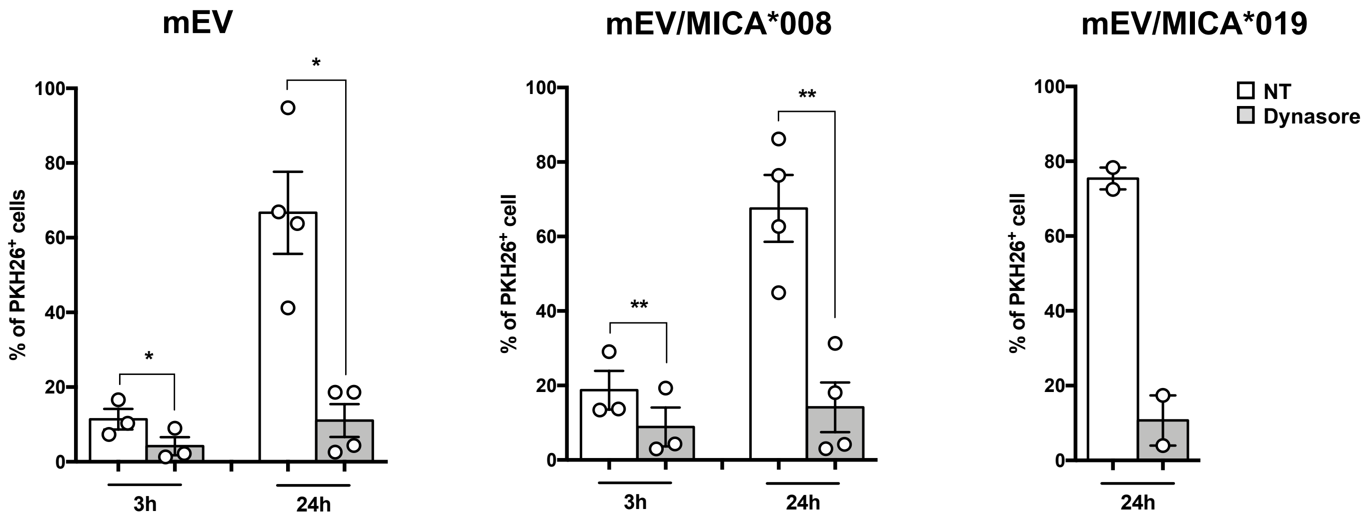

Supplementary Figure S3. mEV uptake by MM cells occurs through an endocytosis-dependent mechanism. The MM LP1 cell line was treated as described in Figure 2, paneld using PKH26-labelled mEVs. The percentage of PKH26^{+}LP1 cells is shown. Values relative to the mean of at least two independent experiments are shown.

.

Patient n.	Sex/age	stage	Ig
1	F/48	Smoldering	IgG-κ
2	M/66	MGUS	IgG-λ
3	F/74	Onset	IgG-λ
4	M/77	Onset	IgG-λ
5	F/70	Relapse	IgG-κ
6	M/46	Onset	IgA-λ
7	F/71	Onset	IgA-κ
8	M/75	Relapse	IgA-λ
9	M/60	Onset	IgA-λ
10	F/79	Onset	IgA-λ
11	M/83	Onset	IgG-κ
12	F/62	MGUS	IgA-κ
13	F/72	Onset	Light chain mieloma

Supplementary Figure S4. Characteristics of MM patients.

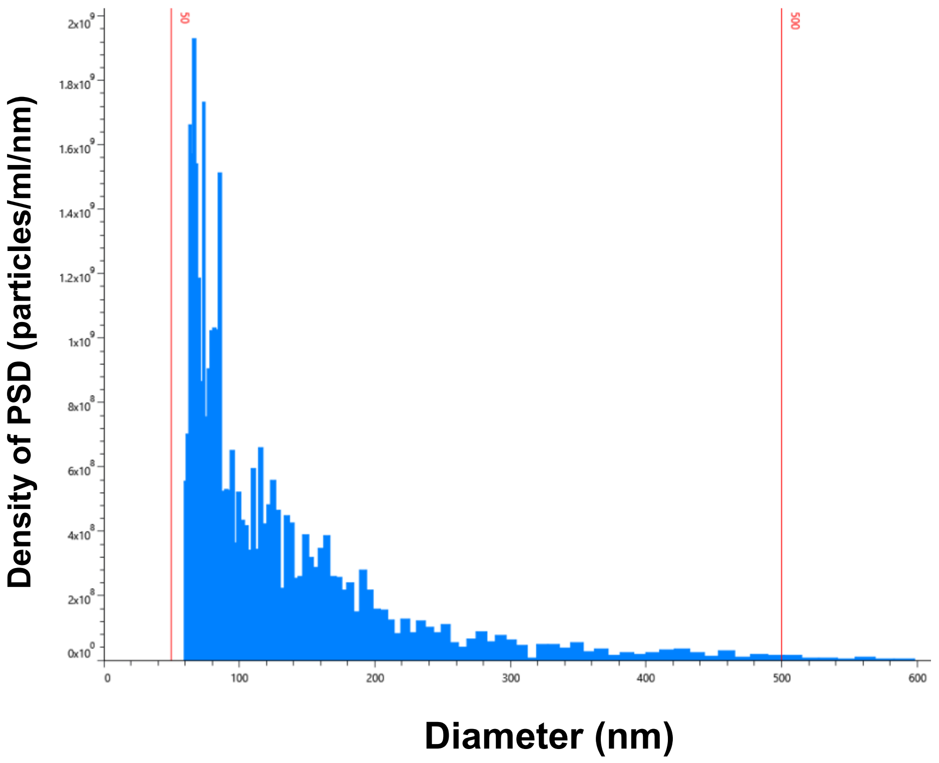

Supplementary Figure S5. Size distribution of mEVs isolated from plasma of MM patients. The size distribution was performed using a ViewSizer^{TM} 3000 (HORIBA Instruments incorporated, Irvine CA, USA). A representative sample derived from BM plasma is shown. The average diameter of EV population corresponds to 140 ± 20 nm.
